# Supplementary figures and images for: Novel metrics reveal new structure and unappreciated heterogeneity in Caenorhabditis elegans development
Source: PLoS Comput Biol. 2023 Dec 19;19(12):e1011733. doi: 10.1371/journal.pcbi.1011733 (PMC10763962; doi:10.1371/journal.pcbi.1011733)

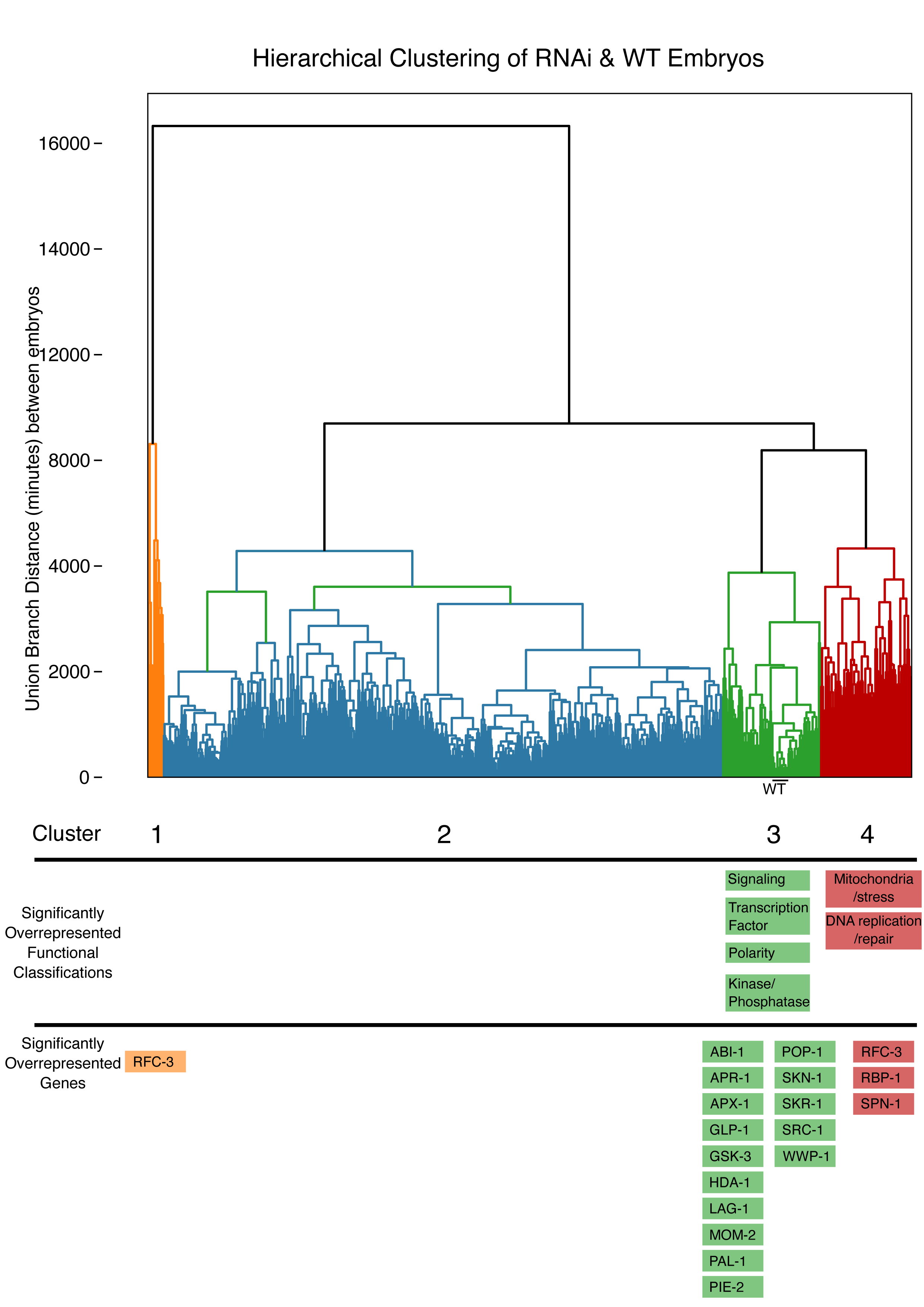

Supplement: S1 Fig — Shown is a dendrogram constructed using the union branch distance measured between all WT and RNAi treated embryos along with the 4 classes we partition the dataset into. The WT embryos and WT-like RNAi-treated embryos are highlighted in cluster 3. Significantly Overrepresented Genes and Functional Classifications in each cluster are listed here. P values were calculated with Boschloo’s test and significance was determined based on a Bonferroni corrected threshold of 1.5 * 10(-4). Raw data are available in supplemental datasets S1 and S3. (TIF) [file pcbi.1011733.s001.tif]

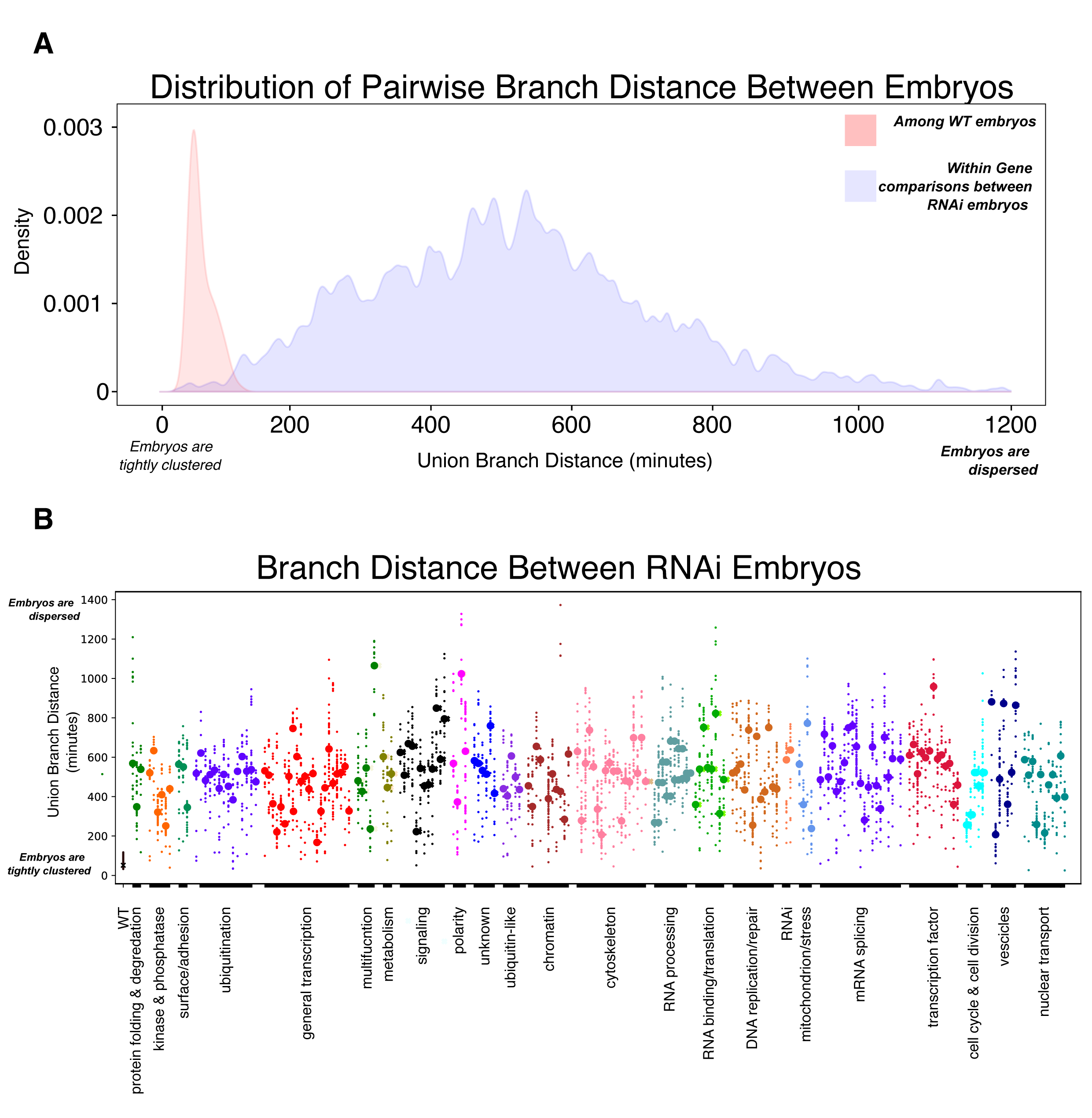

Supplement: S2 Fig — (A) The distribution of union branch distances between wild type embryos (red) and between embryos treated with RNAi against the same gene (blue). Densities were generated using a kernel density estimator. (B) Strip plot of all RNAi embryos showing the branch distances between embryos treated with RNAi against the same gene. Embryos treated with RNAi against genes with shared functions are grouped together. The median branch distance within each set of embryos is plotted as a large circle. (TIF) [file pcbi.1011733.s002.tif]

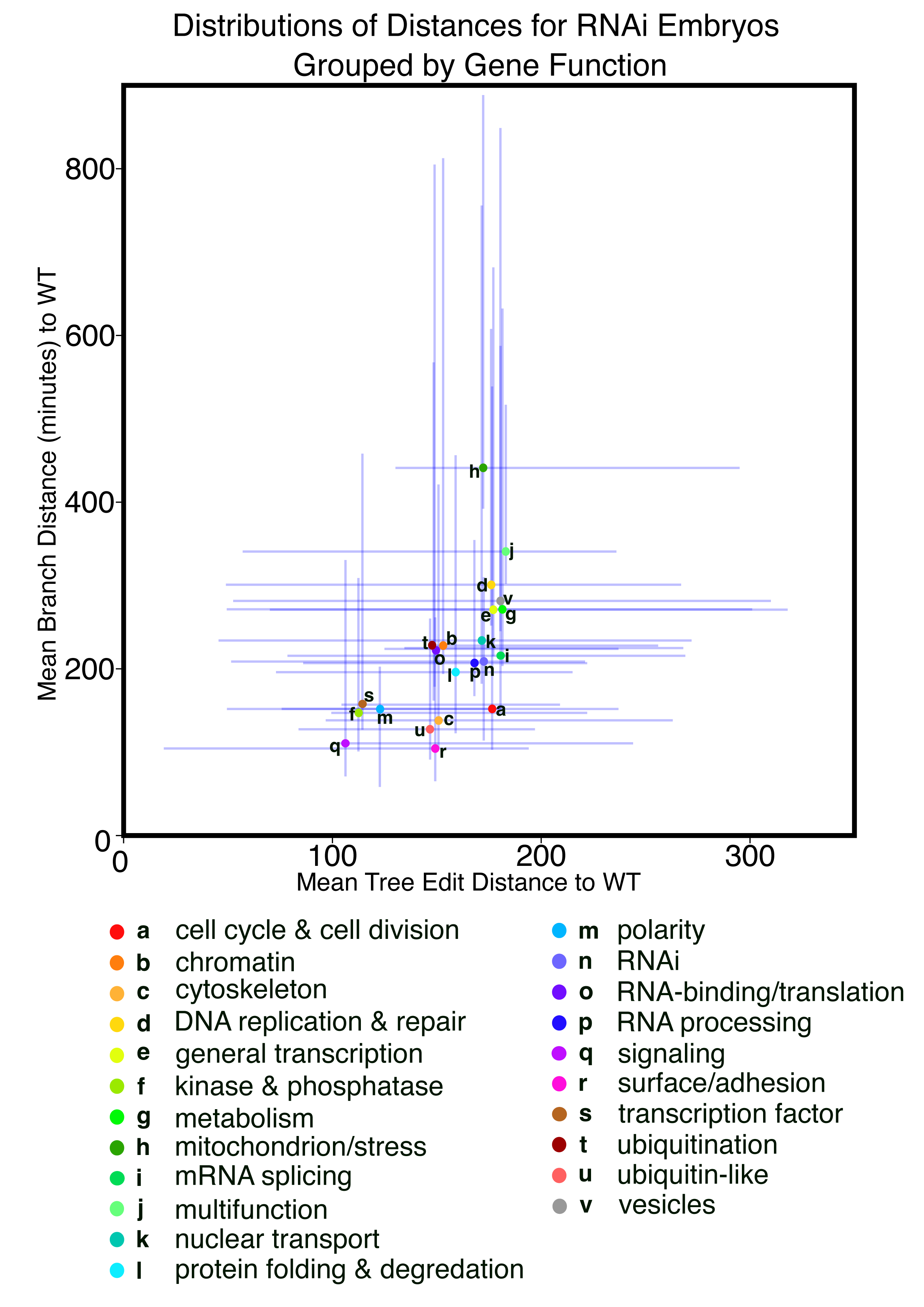

Supplement: S3 Fig — The mean (circle) and minimum/maximum values (blue lines) of the distance between embryos treated with RNAi against genes with common function and a single WT reference embryo are shown. (TIF) [file pcbi.1011733.s003.tif]

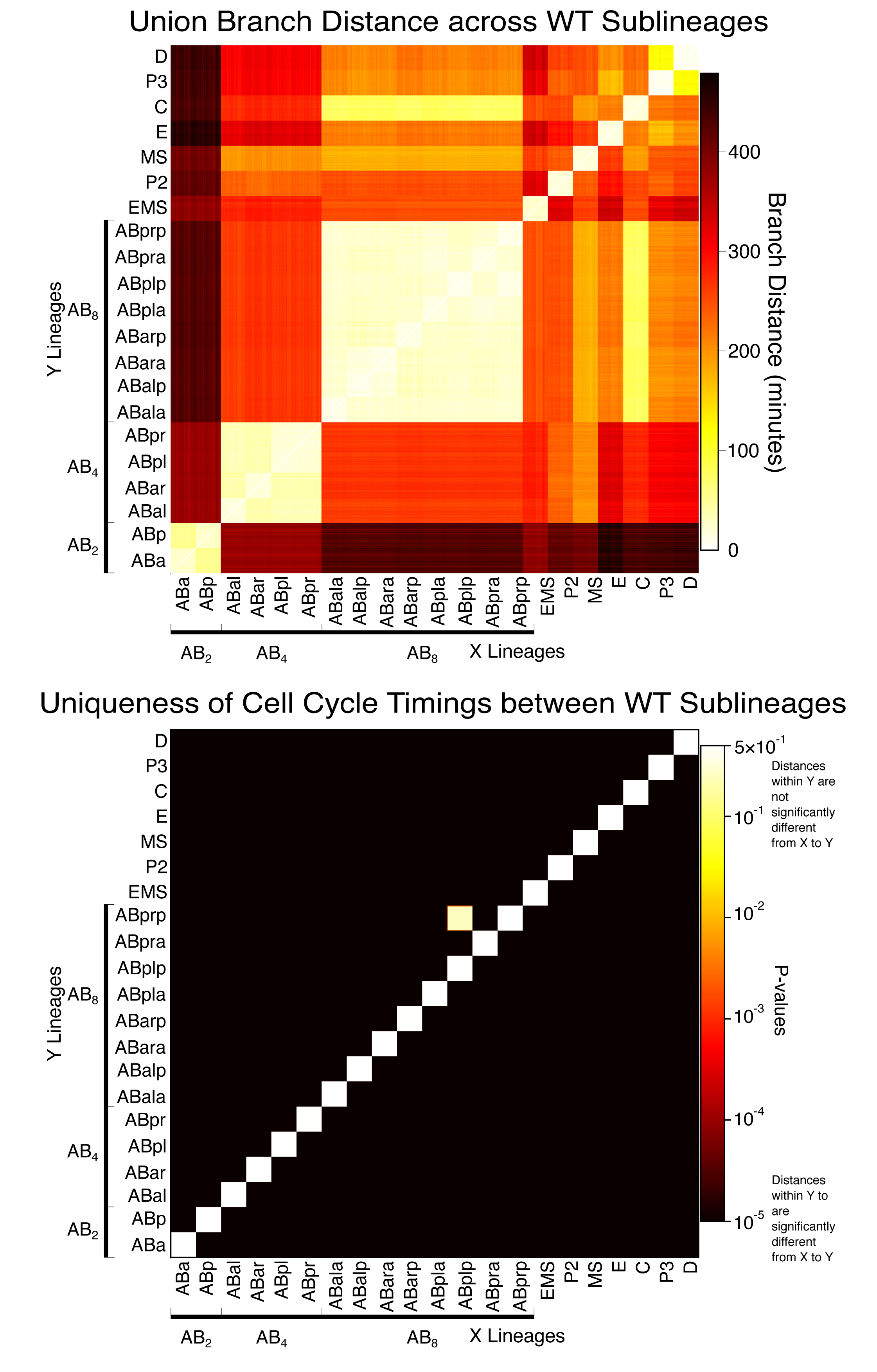

Supplement: S4 Fig — (A) A heatmap showing the union branch distance between each pair of sub-lineages across 21 WT reference embryos. (B) Heatmap showing the p-value calculated using 441 permutation tests (see methods) against the null hypothesis that sublineages share a common distribution of intersection branch distances. All but a single comparison (ABprp vs ABpla) are significant based on a Bonferroni corrected threshold of 2 * 10−5. (TIF) [file pcbi.1011733.s004.tif]

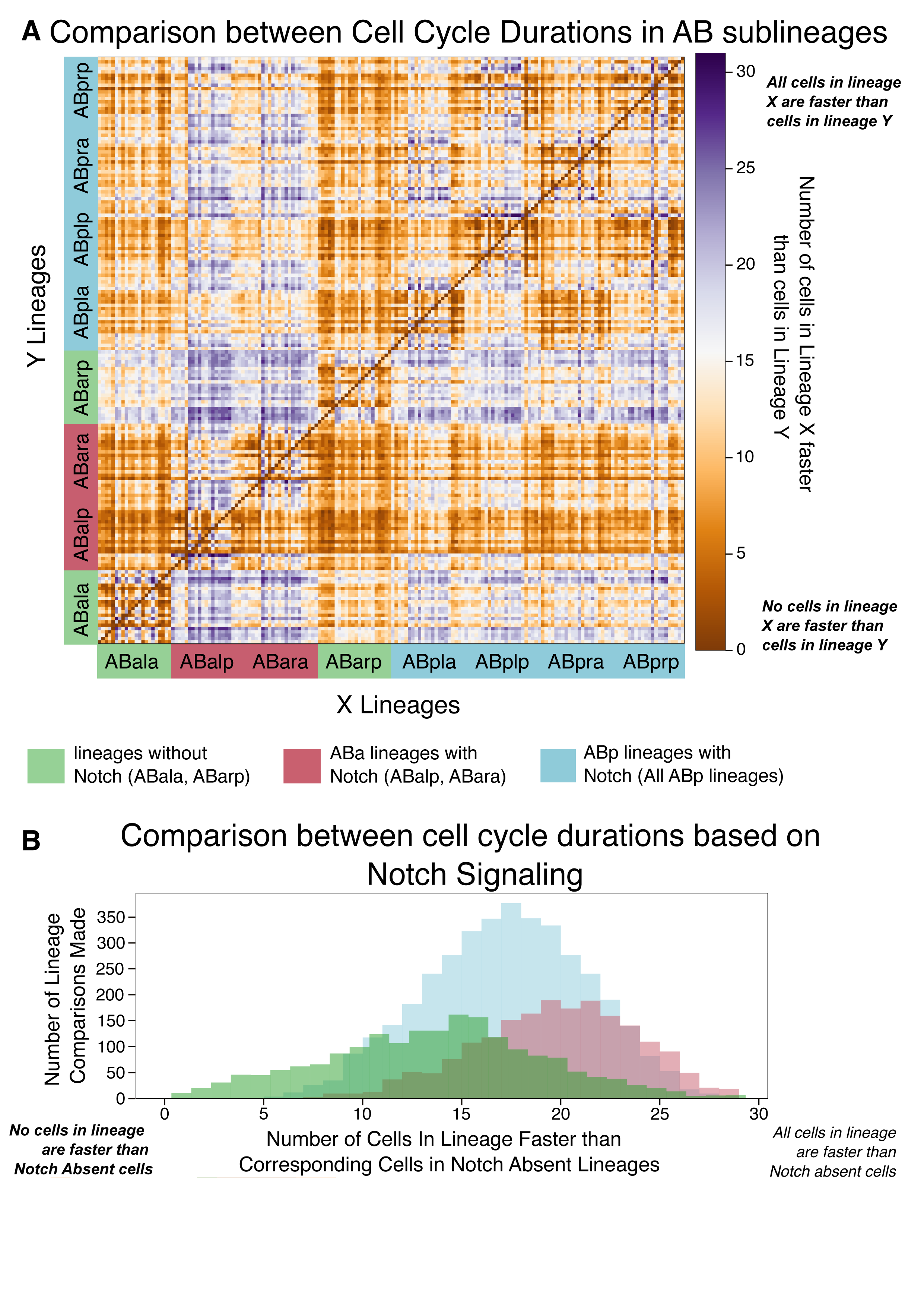

Supplement: S5 Fig — (A) A heatmap showing the fraction of cells in the lineage listed along the Y axis that have a shorter cell cycle than the corresponding cell in the lineage along the X axis. Each sublineage contains 32 cells so a lineage in which >16 cells possess a shorter cell cycle is considered to be “faster” (B) The distribution of comparisons from the heatmap in panel A grouped based on lineages that do not have a history of Notch activation (green, ABala and ABarp), lineages derived from ABa in which Notch is activated (red, ABalp and ABara), and all lineages derived from ABp. (TIF) [file pcbi.1011733.s005.tif]

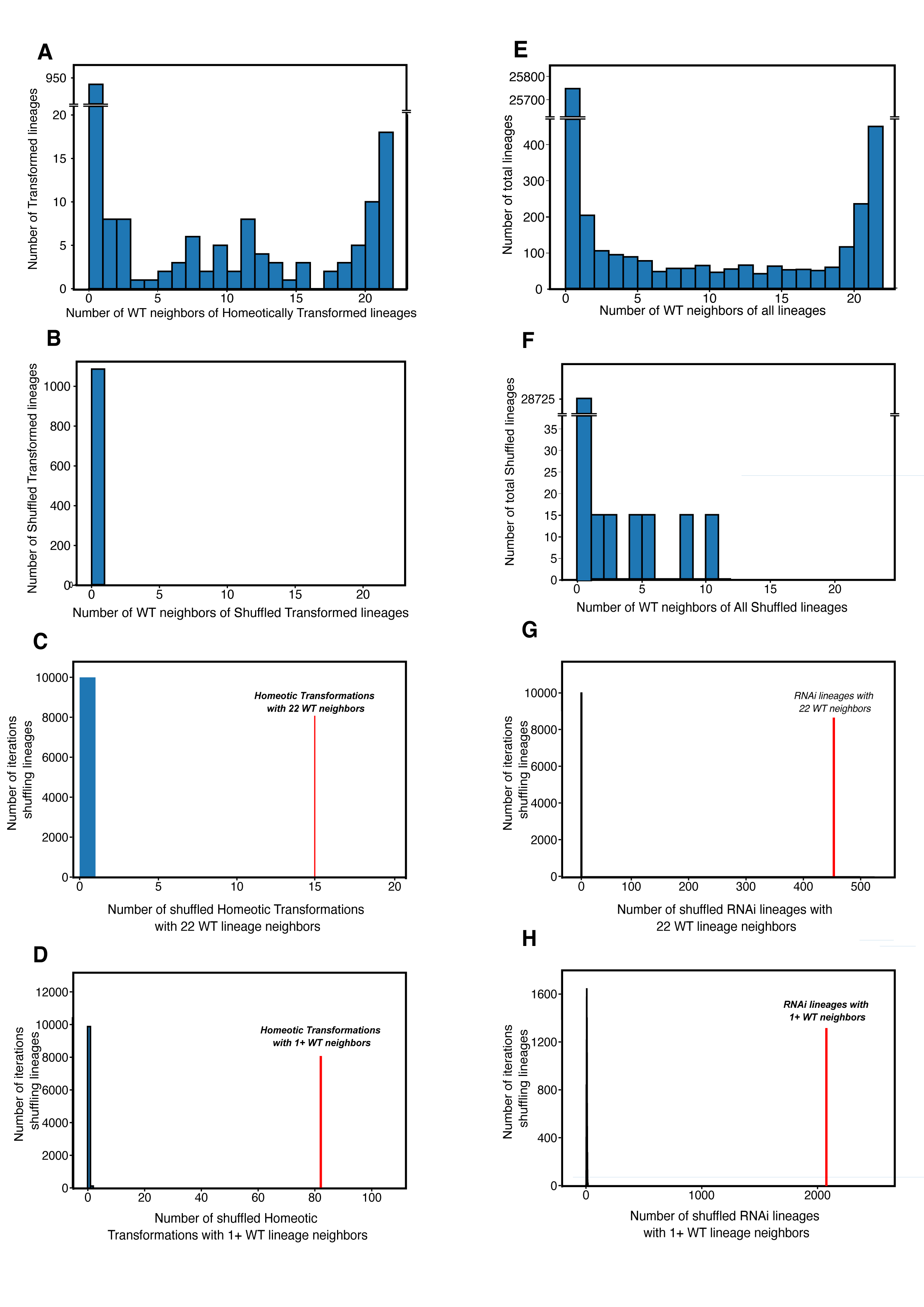

Supplement: S6 Fig — (A) The number of WT destination lineages that fall within the transformed neighborhood of lineage annotated as homeotically transformed by Du et al. [10]. See Main Fig 6B. (B) The number of WT destination lineages that fall within the transformed neighborhood of homeotically transformed lineages where the length of every cell’s cycle is shuffled among all cells of the same name across all embryos treated with RNAi against genes that produce homeotic transformations. Note there are no WT neighbors to any of these shuffled lineages. (C) The number of cases where lineages from RNAi treated embryos that are shuffled as in (B) fall within the neighborhood of all 21 WT samples of the destination lineage. The red line shows the corresponding value for the unshuffled data. (D) The number of cases where lineages from RNAi treated embryos that are shuffled as in (B) fall within the neighborhood of 1 or more WT samples of the destination lineage. The red line shows the corresponding value for the unshuffled data. (E) The number of WT destination lineages that fall within the transformed neighborhood of all sublineages from all RNAi treated embryos. See Main Fig 6C. (F) The number of WT destination lineages that fall within the transformed neighborhood of all lineages from RNAi treated embryos where the length of every cell’s cycle is shuffled among all cells of the same name. (G) The number of cases where lineages from RNAi treated embryos that are shuffled as in (F) fall within the neighborhood of all 21 WT samples of any lineage. The red line shows the corresponding value for the unshuffled data. (H) The number of cases where lineages from RNAi treated embryos that are shuffled as in (F) fall within the neighborhood of 1 or more sample of any lineage. The red line shows the corresponding value for the unshuffled data. (TIF) [file pcbi.1011733.s006.tif]
