# Supplementary material for: Novel metrics reveal new structure and unappreciated heterogeneity in Caenorhabditis elegans development
Source: PLoS Comput Biol. 2023 Dec 19;19(12):e1011733. doi: 10.1371/journal.pcbi.1011733 (PMC10763962; doi:10.1371/journal.pcbi.1011733)
Supplement: S1 Appendix — (PDF) [file pcbi.1011733.s011.pdf]

Appendix

1 Generalizing the Branch Distance

Let T_p be the space of all binary trees of depth p that are rooted, weighted, and perfect. In this context, a perfect tree simply means that all nodes that are neither leaf nodes nor the root node have a degree of exactly 3, and all possible nodes in a binary tree of depth p are present. In other words, such a tree is complete, and has no missing nodes. By weighted, in this context we mean that every node has associated with it a weight $w \in \mathbb{R}$. Formally, we can think of this as a map $W : N \to \mathbb{R}$, where N is the set of nodes in the tree. We use indices to indicate this relationship; for instance, if a node n has associated with it weight w then we write w_n.

Consider two trees $T_1, T_2 \in T_p$, with sets of nodes N_1 and N_2 respectively. To make the discussion below more compact, we will say that the weight of any node $n \in N_1$ is denoted $w_{1,n}$ and the weight of a node $m \in N_2$ is $w_{2,m}$. Let an "alignment" A between the two trees be defined as a bijective mapping of nodes on tree T_1 to nodes in tree T_2, $A : N_1 \to N_2$, with the following restriction. If node $n_1 \in N_1$ is mapped to a node $n_2 \in N_2$, then all the child nodes of n_1 are mapped to child nodes of n_2. Note that this restriction means that the alignment in some sense respects the topology of the two trees. Also note that, since this map is bijective, only nodes of the same "level" or "depth" in the two trees can be aligned to one another.

Let α be the set of all possible alignments that exist between any two in T_p. Note that there are 2^{2p-1} such alignments, in other words, $|\alpha| = 2^{2p-1}$.

In addition, if $\exists A \in \alpha$ such that, $\forall n \in N_1$, $w_{1,n} = w_{2,A(n)}$ then we say $T_1 = T_2$. in other words, if two trees can be aligned such that all the corresponding weights are equal, then the trees themselves are equivalent.

Given $T_1, T_2 \in T_p$ and an alignment $A \in \alpha$, we define a function $D_A : T \times T$ that will form the basis for our metric. To compute D, we first use the alignment A_1 to pair the nodes from the tree with one another. We then define D as:

$$D_A(T_1, T_2) = \left(\sum_{n \in N1} (w_{1,n} - w_{2,A(n)})^2 \right)^{\frac{1}{2}} . \tag{1}$$

Put simply, this is just the normal Euclidean distance between the weights where nodes that are aligned to one another are compared to one another in the distance formula.

To define the distance between two trees in this framework, we put $d : T \times T \to \mathbb{R}$ as:

$$d(T_1, T_2) = min_{A \in \alpha}(D_A(T_1, T_2)). \tag{2}$$

In other words, the distance between two trees is the minimum value of D_A across all possible alignments. We now show that $d(T_1, T_2)$ is a metric by demonstrating that it satisfies the following axioms:

Reflexivity $d(T_1, T_1) = 0$. Choose the trivial alignment $A(n) = n$. Then $w_{1,A(n)} = w_{1,n}$ and $D_A(T_1, T_1) = 0$. Since by definition $D_{Ai}(T_1, T_2) \geq 0$ $\forall T_1, T_2 \in T_p$ and $A_i \in \alpha$, this is the minimum value of D_{Ai} and the conclusion follows.

Positivity If $T_1 \neq T_2$, then $d(T_1, T_2) \geq 0$. Note that, if $T_1 \neq T_2$, by our definition of equality of trees there $\nexists A : \forall n \in N_1,\ w_n = w_{A(n)}$. In other words, there is no alignment that can align nodes such that all the weights are equal under the alignment. This implies $D_A(T_1, T_2) \geq 0\ \ \forall A \in \alpha$, and the conclusion follows.

Symmetry $d(T_1, T_2) = d(T_2, T_1)$. Put A as the alignment that gives the minimum $D_A(T_1, T_2) = d(T_1, T_2)$. Since A is bijective, $D_{A^{-1}}(T_2, T_1) = D_A(T_1, T_2)$, and since $A^{-1} \in \alpha$ the conclusion follows.

Triangle inequality $d(T_1, T_3) \leq d(T_1, T_2) + d(T_2, T_3)$. Take A_1 to be the alignment that gives the minimum value of D for T_1 and T_2, and similarly A_2 to be the alignment that gives the minimum value of D for T_2 and T_3. Put A as the composition of these two alignments, which maps nodes from tree T_1 to nodes in tree T_3. Now clearly $D_A(T_1, T_3) \leq D_{A_1}(T_1, T_2) + D_{A_2}(T_2, T_3)$ by the fact that the definition of D_A is simply the definition of the Euclidean norm in a finite-dimensional vector space over $\mathbb{R}$. Since $d(T_1, T_3) \leq D_A(T_1, T_3)$ by definition, the conclusion follows.

2 Relationship between the Robinson-Foulds and Tree Edit Distance

In this work, we use the Tree Edit Distance to describe topological differences between lineages. While this is a well-established and intuitive metric, other ways of measuring the topological differences between networks have been proposed. One prominent example is the Robinson-Foulds (RF) metric for the difference between two trees with labeled nodes [1]. The RF distance is based on the fact that, since trees are acyclic graphs, if you remove an edge from a tree, you will obviously generate two different components (i.e. at two different clusters of nodes). Since the trees are labeled, this will produce two sets of labels, one for each of the two components that are generated. Given two trees T_1 and T_2, an edge in T_1 can be "matched" with an edge in T_2 if it produces the same two sets of labels in this way. In other words, call the set of edges in T_1 E_1 and the set of edges in T_2 E_2. Then some edge $e_i \in E_1$ is matched with some edge $e_j \in E_2$ if removal of e_i from T_1 produces the same partitioning of labels as removal of e_j from T_2. The RF distance is then simply the number of edges in T_1 that *cannot* be matched with an edge in T_2 plus the number of edges in T_2 that cannot be matched with an edge in T_1 (in other words, just the total amount of unmatched edges between the two trees).

Since the RF distance is an established and well-studied metric on trees, one might ask whether it provides different results from the Tree Edit distance. Interestingly, for the case we consider here, the RF distance and the Tree Edit Distance are equivalent. Note that the only application of the Tree Edit Distance in this work is to compare RNAi trees to the WT tree to determine the extent of topological changes induced by knock-down of the gene in question. Because of the eutelic nature of *C. elegans* development, every RNAi lineage is actually a subtree of the WT lineage.

In our implementation of the Tree Edit Distance, we quantify the topological changes by simply counting the number of nodes from one tree that are absent from another; conveniently, this can be computed as the size of the symmetric difference between the set of nodes in the WT tree and the set of nodes in the RNAi tree. The RF distance, suitably applied to this problem, would naturally count the number of edges that are missing in the smaller of the two trees. Since the trees in question are completely binary, the number of edges removed by removing a subtree is exactly equivalent to the number of nodes removed, since every node must be connected to the tree through an edge to its parent node. So, while the two metrics are not equivalent for all comparisons between tress, for this specific case they are the same.

Another metric that has been developed to quantify the topological differences between trees is the Triplet Distance, which measures the number times a set of three leaf nodes from one tree has a different topology from that same set of three nodes in the other tree [2]. In the case of the *C. elegans* lineage, all cells are labeled based on their lineage relationship, meaning that sibling/cousin relationships are encoded in the labels. Triplets sampled from such labels will

always match the topology shared by those labels in any tree, except in the case of absent cells caused by a missing division, which is the exact difference enumerated by the tree edit distance. The triplets metric is thus not suitable to compare *C. elegans* lineages reconstructed by direct observation where sibling/cousin relationships are empirically accurate, motivating our use of the Tree Edit Distance instead.

References

[1] Robinson, D. F., & ; Foulds, L. R. (1981). Comparison of phylogenetic trees. Mathematical Biosciences, 53(1–2), 131–147. https://doi.org/10.1016/0025-5564(81)90043-2

[2] Sand A, Holt M, Johansen J, Fagerberg R, Brodal G, Pedersen C, et al. Algorithms for Computing the Triplet and Quartet Distances for Binary and General Trees. Biology [Internet]. 2013 Sep 26;2(4):1189–209. Available from: http://dx.doi.org/10.3390/biology2041189
