# Supplementary material for: Novel metrics reveal new structure and unappreciated heterogeneity in Caenorhabditis elegans development
Source: PLoS Comput Biol. 2023 Dec 19;19(12):e1011733. doi: 10.1371/journal.pcbi.1011733 (PMC10763962; doi:10.1371/journal.pcbi.1011733)
Supplement: Attachment — Submitted filename: Review_PLoSComputBiol_ZZ.pdf [file pcbi.1011733.s012.pdf]

The authors introduce a generalized metric to compare lineage topology between wild type (WT) or RNAi embryos. They apply the metric to the data on cell-cycle timing from over 1300 WT and RNAi-treated Caenorhabditis elegans embryos. They claim that they found surprising heterogeneity within this data set, including subtle batch effects in WT embryos and dramatic variability in RNAi-induced developmental phenotypes. They also claim that heterogeneity have been missed in previous analyses. After further analysis of these results, they suggests a novel, quantitative link between pathways that govern cell fate decisions and pathways that pattern cell cycle timing in the early embryo. Overall, the branch distance they proposed has the potential to more accurately quantify the subtle variation/change in lineage topology than the existing ways of lineage quantification. At the same time, there are many issues that needed to be addressed before acceptance for publication.

Major comments

1. The authors claim that heterogeneity in lineage topology is missing previously. However, this claim seems not be able to hold. For example, impact of Notch signaling on cell cycle timing [https://doi.org/10.15252/msb.20145857], variability of cell lineage between wild type embryos [http://doi.org/10.1016/j.ydbio.2012.11.034] among others. They should articulate what the different finding they made compared with previous findings. These previous findings should be properly cited.
2. The method for calculating cell cycle times was reported previously [https://doi.org/10.15252/msb.20145857] and [https://doi.org/10.1101/776062], while it is well justified why the authors used cell cycle time instead of cell division time (although it might be of less importance since this has been used by other groups and for many years). This should be mentioned and critiqued in their introduction.
3. In Figure 3, the branch distance can tell the difference in the developmental pace between two clusters, but this has also been reported by the two pieces of literature above. So it's a support for the authors' method as well, not new finding.
4. In Figure 4, the authors reported the phenotypic heterogeneity within RNAi-treated embryos of the same gene and between RNAi-treated embryos of different genes, which was not unexpected, and the possibility of experimental artifacts (*e.g.*, temperature control) cannot be excluded. It's still difficult to interpret the clusters.
5. In Figure 5, the authors report how Notch signaling affects cell lineage. It would be better to putthe results in the context of what has been reported in [https://doi.org/10.15252/msb.20145857] and what novel finding is here.
6. In Figure 6, although a common genetic architecture controlling cell division asynchrony and cell fate asymmetry was reported, the idea of using cell lineage to predict cell fate is novel and interesting. It should be expanded more regarding its potential to uncover the underlying genetic functions.

Specific Comments:

1. Page 4, Line 71, "*Aligning measurements such as…*" —— Apart from the developmental properties mentioned by

the authors, another well-known hallmark of *C. elegans* development is its stereotypical cell position [https://doi.org/10.1016/0012-1606(83)90201-4]. Aligning measurements and intuitive visualization has been done in [http://doi.org/10.1016/j.ydbio.2012.11.034] and [https://doi.org/10.1101/776062], which should be mentioned to give a more comprehensive description of stereotypical *C. elegans* development.

2. Page 4, Line 83, "*This property dramatically simplifies the application of metrics computed on lineage trees…*" —— [https://doi.org/10.15302/J-QB-022-0319] proposed several graph metrics for *C. elegans* embryonic cell lineage recently and their methods can also tell the difference between the differentiated ABp and EMS blastomeres, so it should be cited.
3. Page 5, Line 105, "*We extended these measurements…*" —— The role of Notch signaling in the control of developmental timing has been reported previously by [https://doi.org/10.15252/msb.20145857] using the wild-type and RNAi-treated embryos collected with similar experimental methods of the embryos used in this manuscript, but was ignored here. The paper reported the effect of *lag-1, sel-8, ref-1,* and *lin-12*/*glp-1* (the gene specifically studied in this manuscript) on the developmental timing in Fig. 5. The authors should at least explicitly describe what is new they found compared to the previous knowledge in this highly relevant literature, and their *Introduction*, *Results*, and *Discussion* parts should be rephrased accordingly.
4. Page 6, Line 129, "*The first is the tree edit distance…*" —— The quantitative comparison between a pair of cell lineages proposed in this manuscript needs to determine the nodes at the beginning, and calculate the three values. The nodes are counted within a time window, but how to establish this time window for different cell lineages? For example, regarding the AB lineage, the wild-type embryos achieve 8 or even more AB cells at different timing, so the operation for picking up the time window must affect the calculation results, especially for the RNAi-treated embryos with diverse cell lineages. The authors should provide thes details in the main text or supplemental material to accommodate these problems.
5. Page 7, Line 160, "*Fig 1…*" —— The mathematical form of equations and symbols should be consistent across the whole manuscript, for example, now the L^2 is italic on Page 7, Line 160 but all the mathematical symbols in Fig. 1 are not. Please also indicate that TED, IBD, and UBD are abbreviations in either the figure or its legend to avoid confusion.
6. Page 7, Line 160, "*Fig 1…*" —— The idea that uses graph metrics to compare any measurements on two cell lineages is interesting. The number noted near the branch is such an assumed measurement, like the cell cycle timing or gene expression level that has been emphasized in this manuscript. However, the figure presentation is a little bit confusing as the branch length is not based on its assumed cell cycle timing and the branch color is not consistent with its assumed gene expression level. The authors may want to present it in a more generalized way, but it is difficult to be understood by the audience. One suggestion is to expand the figure by showing how cell cycle timing or gene expression level is distributed on the cell lineages according to their given values or based on a real case (real cell cycle data and actual gene expression data).
7. Page 8, Line 180, "*While the spatial organization…*" —— The statement "*the distribution of cell cycle times within*

lineages have not been as deeply explored" is inappropriate. There are two pieces of literature, [https://doi.org/10.15252/msb.20145857] and its subsequent update [https://doi.org/10.1101/776062], which calculated this distribution with similar methods (*i.e.*, using cell cycle times instead of cell division times) and produced similar results (*i.e.*, finding the high correlation between cell cycle times of different wild-type embryo samples) as reported in this manuscript. Those previous results should be mentioned for supporting/validating the data and method used in this manuscript and the subtitle "*unexpected batch effects*" should be revised/softened.

8. Page 12, Line 227, "*Surprisingly, clustering on…*" —— Why are there two distinct populations of WT embryos? Do they come from different experimental conditions [https://doi.org/10.1016/j.ydbio.2012.11.034]? If the embryo data is solid, there should be a complete record of this, and the authors should be able to explain why this happen and avoid any possible artificial/methodological mistakes. Otherwise, there might be something wrong in the data used in this manuscript. More importantly, this can help prove the power of the graph metrics.
9. Page 13, Line 249, "*Fig. 4…*" —— Which cluster is the wild-type embryos? Should they be separated as they are supposed to have the highest similarity?
10. Page 14, Line 261, "*We hierarchically clustered these embryos based on the union branch distance into 4 major groups…* " —— As stated above, why can the RNAi-treated embryos be clustered? Do they have different known gene functions? As mentioned by the authors that the graph metrics can tell the difference between the developmental pace of wild-type embryos, is it possible this clustering results just come up because the original experimentalists have different experimental conditions such as a slight change in temperature? The authors should explain the reason or at least, make speculation instead of just drawing strong conclusions with the computational outputs.
11. Page 14, Line 274, "*Surprisingly, this variability isn't a simple manifestation of variable phenotypic severity, as these embryos often differ from one another as much as they differ from wild type…*" —— This comparison of variability is insufficient to claim that it is not variable phenotypic severity. In theory, a multi-body system is sensitive to thermal noise, which is common in a biological system, then the system state would keep being diversified when the noise is not regulated/controlled ~ the so-called butterfly effect in nonlinear science. This has been demonstrated by mechanical simulation with random perturbation previously [Fig. 3D and Fig. 5C of https://doi.org/10.1242/dev.154609; Fig. 3 of https://doi.org/10.1088/1478-3975/ab6356]. Also, the diversified phenotypes within wild-type nematode embryos [Fig. 7 of https://doi.org/10.1242/dev.200401] or RNAi-treated nematode embryos [Fig. S10 of https://doi.org/10.1371/journal.pcbi.1009755] are not surprising, where experimental scientists usually only consider the highly reproducible phenotypes as the unreproducible ones can be from all kinds of unknown reasons.
12. Page 35, Line 708, "*Biophysical parameters such as cell volume affect cell cycle duration…*" —— There are two other pieces of literature that quantitatively measure how cell volume affects cell cycle duration during *C. elegans* embryogenesis [https://doi.org/10.1088/1367-2630/aaea91; https://doi.org/10.1103/PhysRevE.104.054409].
