# Supplementary material for: Novel metrics reveal new structure and unappreciated heterogeneity in Caenorhabditis elegans development
Source: PLoS Comput Biol. 2023 Dec 19;19(12):e1011733. doi: 10.1371/journal.pcbi.1011733 (PMC10763962; doi:10.1371/journal.pcbi.1011733)
Supplement: Attachment — Submitted filename: ResponseToReviewers.pdf [file pcbi.1011733.s013.pdf]

We thank the reviewers for their considered and detailed feedback, their appreciation of our work, and the valuable suggestions that we have incorporated to improve the overall quality of our study and manuscript. Below we have detailed our response to major and minor concerns raised by the reviewers, how we have addressed them experimentally and in the text of the manuscript.

Reviewer 1 Commentary:

Reviewer #1: The authors in this interesting manuscript claim that they have introduced a generalized metric, branch distance, that allows the discovery of new variability based on phenotypic measurements in individual cells during the development of C. elegans embryos. My main concern with this study is

Comment 1:

1) the lack of proven generalizability of the metric, as it seems to me that it can only for now be applied to organisms with a very well defined cell lineage such as C elegans.

We thank the reviewer for raising an extremely important concern regarding the generalizability of the branch distance that we defined. The reviewer is absolutely correct that the definition we used in our original manuscript relies heavily on the fact that there is a deterministic naming convention for cells in the developing *C. elegans* embryo. This makes it trivial to determine that the cell cycle time of one cell in one embryo corresponds to the cell cycle time of that same cell in a different embryo, allowing us to naturally “align” any two trees corresponding to two different embryos. This metric is indeed generalizable to cases where no such naming convention exists. We have detailed that generalization below and in the revised version of the manuscript.

Generalizing the branch distance to unlabeled binary trees reveals robustness in WT lineages

A key component of our branch distance metric involves aligning the binary trees being compared; this just means determining which cell in the first tree (call this tree T_1) corresponds to which cell in the second tree (tree T_2). In our original manuscript, the individual labels make this alignment trivial; the cell ABa from tree T_1 clearly corresponds to ABa from tree T_2. The question then becomes, how do we perform such an alignment when there is no natural correspondence between cells in the two trees? There are several possible ways to make this definition, but we felt that the most natural is to consider all possible alignments between the two trees, and define the distance based on the alignment that results in the *minimum* branch distance. Note that there are several key constraints on this alignment. For one, we require that nodes only be aligned to nodes of the same “level” in the other tree; in other words, a leaf node of one tree can’t be aligned with the root node of the other tree. Secondly, if a node in one tree is aligned to a node in another, the child nodes must be aligned with one another as well. In other words, say we align node X from tree T_1 to node Y in tree T_2. Then the child nodes of X can only be aligned to the child nodes of Y. This ensures that the alignments respect the topological structure of the tree.

The idea behind this metric is that there are a set of alignments that satisfy these constraints, and we choose the alignment that provides the smallest branch distance. In some sense, this is similar to the way that geodesic distances on manifolds are defined: we define the geodesic distance to be the minimal (technically infimum) distance across all the paths between the points on the manifold. This definition is discussed in more technical detail in the appendix of the revised supplementary information. While this definition can be generalized to cases where the binary trees are not complete

(i.e. when some nodes are “missing” from the tree), here we focus on the case of complete binary trees to simplify the analysis.

Unfortunately, there are no available datasets of which we are aware that could be used to test this generalized version of the metric in species without deterministic cell labels in the lineage tree. We thus benchmarked our generalized metric using the WT *C. elegans* AB lineage. This lineage always corresponds to a complete binary (sub-) tree in the WT data, simplifying the application of our generalized metric. To perform benchmarking, we removed all the labels of the nodes and performed an exhaustive search over all possible alignments and found the one that gave the minimum branch distance (based on cell cycle times) between any two WT embryos.

The results of application of the generalized branch distance can be found in Figure 7 of the revised main text (page 18), which is copied below. Essentially, the patterns that we found through application of the generalized branch distance are very similar to the ones we found using the original branch distance, although of course distances are smaller in the generalized case (Figure 7A vs. 7B). To highlight the extent to which this approach changes our result, we also calculated how many differences there were between the original alignment (based on canonical cell labels, e.g., Figure 7A) and the new alignment (Figure 7B). In other words, we counted the number of cases in which cells were *not* mapped to their canonical partners in this new alignment that gives the global minimum distance (Figure 7C). Interestingly, these findings suggest more variation in some sublineages than in others.

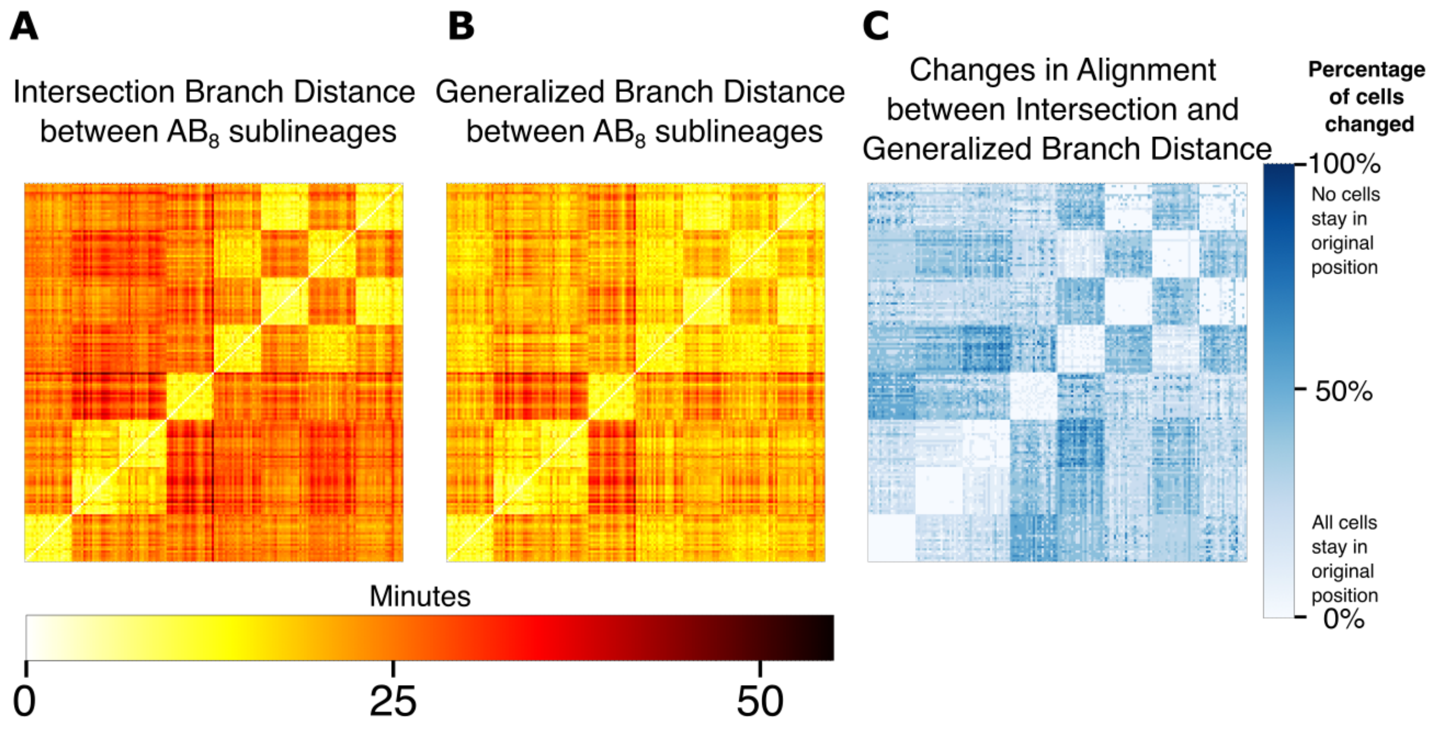

These findings demonstrate that the branch distance we define is readily generalizable. We note that there are a number of limitations to the generalization presented in the revised manuscript: for instance, the current generalized metric is only applicable to complete binary trees. Also, alignment of trees is computationally expensive. In this case we did an exhaustive search, which was sufficiently computationally efficient to handle trees with up to 5 levels. Trees with many more levels will necessitate the application of more advanced algorithms, based, e.g., on dynamic programming, in order to more efficiently calculate the minimum distance. We feel that extending the definition, and

designing efficient algorithms with which to apply it, are well beyond the scope of the current work. That being said, the above demonstrates that the metric we define here is in no way restricted to cases with deterministic cell labels. New results relating to the generalized version of our metric may be found on pages 18 to 19 of the main text. We feel that the addition of these results have significantly improved the manuscript, so we thank the reviewer for their excellent suggestion.

Comment 2:

2) Although the discovery of variability in the phenotype made by comparing close cells in the lineage tree (using their branch distance metric) using their phenotypic measurements (i.e gene expression and cell division time) are interesting, the authors need to prove that other used metrics such as RF or triplets would not give similar results.

The reviewer raises a very interesting question that our original manuscript did not adequately address. To summarize, we discuss two different types of metrics in our manuscript. The first metric is an adaptation of the Tree Edit Distance, a metric in graph theory that measures topological differences in trees by counting the number of nodes that need to be added/subtracted to turn one tree into another. Interestingly, when applied to rooted, labeled binary trees, the Robinson Foulds (RF) metric and the tree edit distance are essentially equivalent. Specifically, in our case we only apply the Tree Edit Distance to measure the difference between WT and RNAi lineages. Our Tree Edit Distance quantifies the number of cells that are missing between the RNAi tree and the WT tree. In this context, the RF distance would measure the number of missing edges. Any cell that is in the WT lineage but not in an RNAi lineage would entail “losing” the edge to that cell, and all of the edges of the descendants. Since every cell that is not the zygote (i.e., every node on that is not the root of the tree) has exactly one edge to its “parent” cell, these two numbers are equivalent. We should note that this is not true *in general*, but is true in our specific case.

The triplet distance, on the other hand, measures differences between labeled rooted trees by listing all subtrees with three leaves in each tree and counting the number of triplets with disagreeing topologies. In the case of the *C. elegans* lineage, all cells are labeled based on their lineage relationship, meaning that sibling/cousin relationships are encoded in the labels. Triplets sampled from such labels will always match the topology shared by those labels in any tree, except in the case of absent cells caused by a missed division, which is the exact difference enumerated by the tree edit distance. The triplets metric is thus not suitable to compare *C. elegans* lineages reconstructed by direct observation where sibling/cousin relationships are empirically accurate, motivating our use of the Tree Edit Distance instead.

The branch distance, on the other hand, is a generalization of the Euclidean norm to the space of weighted and labeled tree graph structures. As the other listed metrics measure and operate on tree topology, we note that branch distance is not comparable to these metrics, as it operates on the weights of the nodes. In other words, the metrics that the reviewer mentioned as potential benchmarks measure different properties than the branch distance and as such cannot be directly compared. To our knowledge, and upon examination of the literature, we find no other previously demonstrated examples of metrics that are similar to the branch distances that we define.

We discuss the relationship between the Tree Edit Distance and the RF distance briefly in the revised supplement in the appendix and in the main text on line 150 of page 5 of the revised manuscript.

If such is the case, their approach would be generalizable to other situations where the lineage tree is not prototypic (the limited case of C elegans) such as for the development of other organisms where both lineage and gene expression can now be traced molecularly. If the authors are unable to show this, nor able to find the genetic origins of the correlations/diversity between cell division variability and cell lineage status, this study would be of restricted interest.

Reviewer 2 Commentary:

Reviewer #2: The authors introduce a generalized metric to compare lineage topology between wild type (WT) or RNAi embryos. They apply the metric to the data on cell-cycle timing from over 1300 WT and RNAi-treated Caenorhabditis elegans embryos. They claim that they found surprising heterogeneity within this data set, including subtle batch effects in WT embryos and dramatic variability in RNAi-induced developmental phenotypes. They also claim that heterogeneity has been missed in previous analyses. After further analysis of these results, they suggest a novel, quantitative link between pathways that govern cell fate decisions and pathways that pattern cell cycle timing in the early embryo. Overall, the branch distance they proposed has the potential to more accurately quantify the subtle variation/change in lineage topology than the existing ways of lineage quantification. At the same time, there are many issues that need to be addressed before acceptance for publication.

Major Comment 1:

1. The authors claim that heterogeneity in lineage topology is missing previously. However, this claim seems not be able to hold. For example, impact of Notch signaling on cell cycle timing [https://doi.org/10.15252/msb.20145857][C1] variability of cell lineage between wild type embryos [http://doi.org/10.1016/j.ydbio.2012.11.034] [C2] among others. They should articulate the different findings they made compared with previous findings. These previous findings should be properly cited.

We would like to thank the reviewer for their detailed feedback. Our initial writing was too narrow in our treatment of the literature and thus failed to properly discuss prior work on cell cycle regulation in the *C. elegans* lineage and have incorporated additional discussion into our manuscript, including incorporating discussion of the subset of the reviewer's suggested references that were previously not included.

We apologize for the lack of clarity regarding our claim of unappreciated heterogeneity in the *C. elegans* lineage. We intended to refer specifically to inter-embryo variability in the wild type embryos characterized by Du *et al* and our discovery of apparent batching in this population based on the branch distance, a feature of these embryos that was not previously recognized owing to the use of correlation-based analyses. Regarding suggested references [C1] and [C2] , the extensive work on *C. elegans* developmental regulation and lineage properties by the Zhao and Murray labs has shed significant light on diverse processes in the embryo. Reference [C1] evaluated comparisons between pairs of sibling cells in the timing of their divisions and found many genes, including Notch, were required for sibling cells to exhibit asynchrony, which was inferred to be a marker of fate asymmetry between siblings. Our study provides a distinct but complementary perspective to this prior work, demonstrating that patterns in cell cycle timing are recognizable between entire sub-lineages of the embryo. We demonstrate the impacts of Notch signaling on divisions across entire Notch-induced lineages, and that Notch-dependent fate asymmetries (lineages rooted at ABp, or at ABalp/ABara) are reflected in cell cycle times across the entire lineage. We have expanded our discussion of our findings in the manuscript to

better contextualize them in light of the prior literature, on line 197 of age 7 and line 428 of page 17 of the revised manuscript.

Regarding [C2], we have expanded our discussion to this previous characterization of variability in later rounds of cell division in embryonic development, beyond those traced in the Du *et al.* dataset that we used to benchmark the branch distance and we have included discussion of this important prior work in our manuscript, on line 784 of page 25 of the revised manuscript.

Major Comment 2:

2. The method for calculating cell cycle times was reported previously [https://doi.org/10.15252/msb.20145857][C1] and [https://doi.org/10.1101/776062][C3], while it is well justified why the authors used cell cycle time instead of cell division time (although it might be of less importance since this has been used by other groups and for many years). This should be mentioned and critiqued in their introduction.

We thank the reviewer for highlighting this important reference in the literature on *C. elegans* lineage analysis. Others have previously performed linear regressions of both cell cycle times and cell birth/division times and used the coefficient of determination and scaling parameters of these regressions to measure developmental coordination, which we have referenced in line 105 of page 4 of the revised manuscript.

Our goal in Figure 2 was to demonstrate and measure the effects of this choice by comparing cell cycle timing and cell birth/division timing regressions using bootstrapping based on lineage shuffling, an analysis which has not previously been explicitly performed in the prior literature. We have thus referenced the prior use of cell timing regressions along with explaining our rationale for the analysis in Figure 2.

Major Comment 3:

3. In Figure 3, the branch distance can tell the difference in the developmental pace between two clusters, but this has also been reported by the two pieces of literature above. So it's a support for the authors' method as well, not a new finding.

As the reviewer notes, previous works have quantified differences in global scaling between *C. elegans* embryos, typically using scaling parameters derived from regression analysis. Our goal in examining this for the WT embryos in the Du *et al.* data was not to claim a novel finding in this regard but to explain the differences between otherwise apparently homogeneous pools of embryos that we discovered using the branch distance. More specifically, we note that the changes reflected by the branch distance capture both scaling and interlineage variability, while regression based methods are more limited in application. We appreciate the notice and have clarified this point in the revised manuscript on line 235 in page 8 and line 261 of page 9.

Major Comment 4:

4. In Figure 4, the authors reported the phenotypic heterogeneity within RNAi-treated embryos of the same gene and between RNAi-treated embryos of different genes, which was not unexpected, and the possibility of experimental artifacts (e.g., temperature control) cannot be excluded. It's still difficult to interpret the clusters.

The reviewer is correct that care must be taken in interpreting clusters among the RNAi-treated embryos in light of the lack of technical control over environmental variables that might influence relative scaling as we discovered among the population of WT embryos. We do note a few insights gleaned from this analysis that we believe are warranted, with some caveats that we address in the manuscript. First, the differences in branch distance we measure between RNAi-treated embryos are notably larger in magnitude than between the two WT clusters, suggesting that differences relative to WT are likely to be reliable reflections of the underlying biology of the embryos. Second, similar to the batch effects among the WT embryos, we observe that the RNAi-treated embryos for most genes were imaged close together in time, increasing the likelihood that environmental effects are unlikely to have been drastically different between multiple embryos of the same perturbation. Based on these observations, we argue that a broad-strokes interpretation of clustering the RNAi-treated embryos is admissible, namely that RNAi-treated embryos in Cluster 3 are relatively similar to each other and to WT embryos, embryos in Cluster 2 are more heterogeneous than Cluster 3 and more dissimilar from WT, while Clusters 1 and 4 are both drastically more heterogeneous and a larger departure from WT. We complement these observations with a gene enrichment analysis where we find that transcription factors, kinase/phosphatases, and genes with functions related to signaling and polarity are more likely to be found in cluster 3 near the WT. These results are discussed in the revised main text on page 11, beginning on line 319.

Major Comment 5:

5. In Figure 5, the authors report how Notch signaling affects cell lineage. It would be better to put the results in the context of what has been reported in [https://doi.org/10.15252/msb.20145857] [C1] and what novel finding is here.

We recognize that [C1] is a highly relevant and significant study that clarifies the relationship between RNAi induced homeotic transformations and corresponding changes in cell cycle time on a cell by cell basis. Specifically, [C1] characterized the contribution of Notch signaling (in addition to other pathways) to asynchronous cell divisions between siblings (which they term ADS).

In [C1], Four Notch related genes were knocked down and the ADS change in certain cells was measured, with a focus on cells in the AB_{64} stage that were notch targets. In particular, knocking down lag-1 resulted in significant changes to all measured notch receptor cells while glp-1 knockdown resulted in significant changes to most notch receptor cells.

We have found that knocking down lag-1 and glp-1 induces cell cycle time patterned homeotic transformations of lineages upstream of the chosen notch targets. We have expanded on this point further in the revised manuscript on line 375 of page 14.

Major Comment 6:

6. In Figure 6, although a common genetic architecture controlling cell division asynchrony and cell fate asymmetry was reported, the idea of using cell lineage to predict cell fate is novel and interesting. It should be expanded more regarding its potential to uncover the underlying genetic functions.

We thank the reviewer for their feedback; we have added additional discussion on line 839 of page 26 on the use of cell lineage-resolved phenotypic measurements for the prediction of cell fate.

Specific Comment 1:

1. Page 4, Line 71, “Aligning measurements such as…” —— Apart from the developmental properties mentioned by the authors, another well-known hallmark of C. elegans development is its stereotypical cell position [https://doi.org/10.1016/0012-1606(83)90201-4][C4]. Aligning measurements and intuitive visualization has been done in [https://doi.org/10.1101/776062] [C3] , which should be mentioned to give a more comprehensive description of stereotypical C. elegans development.

We recognize that the development of *C. elegans* is heavily stereotyped in regards to cell shape, size, migration and patterns of division, along with gene expression, chromatin accessibility, and the effects of genetic perturbations as described by [C3]. We also recognize that each cell is stereotyped in regard to position in the developing embryo.

Because *C. elegans* is a eutelic organism with an invariant developmental lineage [C4], with heavy characterization of the variance present in the biophysical quantities of each cell in the developmental lineage [10.1016/j.ydbio.2008.02.054] [https://doi.org/10.3389/fphys.2014.00529] [https://doi.org/10.1088/1367-2630/aaea91; https://doi.org/10.1103/PhysRevE.104.054409]. [https://doi.org/10.3389/fphys.2014.00529] allowing us an ample base of knowledge upon which to expand. These edits have been added to line 72 of page 3 of the revised main text.

Specific Comment 2:

2. Page 4, Line 83, “This property dramatically simplifies the application of metrics computed on lineage trees…” —— [https://doi.org/10.15302/J-QB-022-0319] proposed several graph metrics for C. elegans embryonic cell lineage recently and their methods can also tell the difference between the differentiated ABp and EMS blastomeres, so it should be cited.

While we were able to locate an abstract for this work in an EBSCO database, the original manuscript itself appears to no longer be indexed by Quantitative Biology, does not appear in their search tool or issue table of contents and the DOI does not resolve. Searching the title or the abstract also does not turn up a full text or valid reference for this work.

Specific Comment 3:

3. Page 5, Line 105, “We extended these measurements…” —— The role of Notch signaling in the control of developmental timing has been reported previously by [https://doi.org/10.15252/msb.20145857] [C1] using the wild-type and RNAi-treated embryos collected with similar experimental methods of the embryos used in this manuscript, but was ignored here. The paper reported the effect of lag-1, sel-8, ref-1, and lin-12/glp-1 (the gene specifically studied in this manuscript) on the developmental timing in Fig. 5. The authors should at least explicitly describe what is new they found compared to the previous knowledge in this highly relevant literature, and their Introduction, Results, and Discussion parts should be rephrased accordingly.

We thank the reviewer for pointing out this connection to prior work. Paper [C1], analyzed Asynchrony in division time between the daughters of specific cells (ADS). After knocking down specific developmental regulators including notch related homologs lag-1, sel-8, ref-1, and lin-12/glp-1, the significance in change of ADS of specific cells were analyzed. Lag-1 knockdown induces significant changes in all 6 notch target cells (ABalpaap, ABplpapp, ABplaaap, ABalppa, ABaraaaa, and ABarapaa) and Glp-1 knockdown produces significant ADS change in 4 of 6 notch target cells (ABalpaap, ABalppa, ABaraaaa, and ABarapaa).

We found that changes in ADS induced by Notch related gene knockdown in specific notch target cells chosen in[C1]are found to have corresponding cell cycle time patterned homeotic transformations of lineages upstream to the chosen notch targets. This has been mentioned in line 109 of page 4, line 466 of page 17, and line 802 of page 25 of the revised manuscript.

Specific Comment 4:

4. Page 6, Line 129, “The first is the tree edit distance…” —— The quantitative comparison between a pair of cell lineages proposed in this manuscript needs to determine the nodes at the beginning, and calculate the three values. The nodes are counted within a time window, but how to establish this time window for different cell lineages? For example, regarding the AB lineage, the wild-type embryos achieve 8 or even more AB cells at different timing, so the operation for picking up the time window must affect the calculation results, especially for the RNAi-treated embryos with diverse cell lineages. The authors should provide these details in the main text or supplemental material to accommodate these problems.

We appreciate the feedback regarding the preprocessing of the dataset. We are using a public dataset of previously published *C. elegans* tracking data (digital-development.org), which contains curated WT and RNAi lineage data and corresponding times.

The raw data provided by Du *et al.* was either truncated based on a specific generation at which they stopped curating the lineage or this point was documented in their supplemental data. We followed the convention of treating all cells present within each tree as being “valid” if they fell within the range specified by Du *et al.* This preprocessing is provided in our codebase, and we have clarified this in the description of our methods, on line 548 of page 20.

Specific Comment 5:

5. Page 7, Line 160, “Fig 1…” —— The mathematical form of equations and symbols should be consistent across the whole manuscript, for example, now the L2 is italic on Page 7, Line 160 but all the mathematical symbols in Fig. 1 are not. Please also indicate that TED, IBD, and UBD are abbreviations in either the figure or its legend to avoid confusion.

In order to help keep track of notation, we have taken this advice into consideration and ensured that “L2” is consistently italicized in all uses. In addition, we included the abbreviations for Tree Edit Distance, Intersection Branch Distance, and Union Branch Distance as TED, IBD, and UBD respectively in Figure 1.

Specific Comment 6:

6. Page 7, Line 160, “Fig 1…” —— The idea that uses graph metrics to compare any measurements on two cell lineages is interesting. The number noted near the branch is such an assumed measurement, like the cell cycle timing or gene expression level that has been emphasized in this manuscript. However, the figure presentation is a little bit confusing as the branch length is not based on its assumed cell cycle timing and the branch color is not consistent with its assumed gene expression level. The authors may want to present it in a more generalized way, but it is difficult to be understood by the audience. One suggestion is to expand the figure by showing how cell cycle timing or gene expression level is distributed on the cell lineages according to their given values or based on a real case (real cell cycle data and actual gene expression data).

We appreciate the feedback regarding the clarity of Figure 1. To address this, we added a color code to figure 1, noting that purple nodes are shared between both embryos, and that red and blue colored nodes are exclusive to embryo X and Y respectively. In addition, we specified that the numbers associated with each cell corresponded to an arbitrary weight. Finally, we changed the label from "Embryo X/Y" to "Tree X/Y" to emphasize the generality of the technique to tree data structures.

Specific Comment 7:

7. Page 8, Line 180, "While the spatial organization…" —— The statement "the distribution of cell cycle times within lineages have not been as deeply explored" is inappropriate. There are two pieces of literature, [https://doi.org/10.15252/msb.20145857] [C1] and its subsequent update[https://doi.org/10.1101/776062] [C3], which calculated this distribution with similar methods (i.e., using cell cycle times instead of cell division times) and produced similar results (i.e., finding the high correlation between cell cycle times of different wild-type embryo samples) as reported in this manuscript. Those previous results should be mentioned for supporting/validating the data and method used in this manuscript and the subtitle "unexpected batch effects" should be revised/softened.

We note that the distribution of cell cycle times has been explored before in literature, particularly in [C1] and its follow up [C3]. Furthermore, we also recognize that the prior established methods of looking at cell cycle time, the coefficient of correlation, and analyzing the scaling of the regression, have been in frequent use. We have thus edited the manuscript on line 188 of page 6 to reflect this and clarified the differences between these approaches and our formulation of the Branch Distance.

Specific Comment 8:

8. Page 12, Line 227, "Surprisingly, clustering on…" —— Why are there two distinct populations of WT embryos? Do they come from different experimental conditions [https://doi.org/10.1101/776062] [C3]? If the embryo data is solid, there should be a complete record of this, and the authors should be able to explain why this happens and avoid any possible artificial/methodological mistakes. Otherwise, there might be something wrong in the data used in this manuscript. More importantly, this can help prove the power of the graph metrics.

There are two distinct populations of WT embryos where 9 embryos have faster cell cycle times than the other 21 embryos. The embryos were raised in standard lab conditions, with the more quickly growing embryos growing on specific days. However, certain embryos in the larger, slower group were also imaged during those days, insinuating that these batch effects were caused by factors that are inherent to the organisms, such as maternal effects, and likely not ambient temperature which would have affected all embryos imaged during the same experiment uniformly. We have clarified this in the revised manuscript on line 255 of page 9.

Specific Comment 9:

9. Page 13, Line 249, "Fig. 4…" —— Which cluster is the wild-type embryos? Should they be separated as they are supposed to have the highest similarity?

We have taken the advice of this reviewer into consideration and listed the presence of the WT embryos in Figure 4A. The WT embryos do have the highest degree of similarity but are embedded in the RNAi space very close to a group of RNAi treated embryos. The presence of WT provides a useful

internal reference to measure the variation of RNAi embryos, but we have modified the figure to make the WT embryos within the dataset more obvious.

Specific Comment 10:

10. Page 14, Line 261, “We hierarchically clustered these embryos based on the union branch distance into 4 major groups… ” —— As stated above, why can the RNAi-treated embryos be clustered? Do they have different known gene functions? As mentioned by the authors that the graph metrics can tell the difference between the developmental pace of wild-type embryos, is it possible this clustering results just come up because the original experimentalists have different experimental conditions such as a slight change in temperature? The authors should explain the reason or at least, make speculation instead of just drawing strong conclusions with the computational outputs.

We appreciate the reviewer’s concern regarding overinterpretation of clustering within the RNAi datasets. While batch effects such as we observed in the WT population are possible, it is useful to note that differences in branch distance between the WT clusters are significantly smaller than differences between RNAi-treated embryos and the WT. Similarly, we observe from the original Du *et al.* dataset that most embryos treated with RNAi against the same gene were imaged on the same or adjacent days, further reducing the risk that large changes in ambient environment would be a major driving force behind intragene differences in branch distance. We incorporate further discussion of these considerations into the manuscript. We build on this clustering by performing a geneset and gene ontology-based enrichment analysis of these clusters and find that the less variable clusters among the RNAi treated embryos are enriched for specific genes and gene functions. We have integrated this enrichment analysis into revised supplemental figure 1 and discuss it in the manuscript on line 327 of page 12.

Specific Comment 11:

11. Page 14, Line 274, “Surprisingly, this variability isn’t a simple manifestation of variable phenotypic severity, as these embryos often differ from one another as much as they differ from wild type…” —— This comparison of variability is insufficient to claim that it is not variable phenotypic severity. In theory, a multi-body system is sensitive to thermal noise, which is common in a biological system, then the system state would keep being diversified when the noise is not regulated/controlled ~ the so-called butterfly effect in nonlinear science. This has been demonstrated by mechanical simulation with random perturbation previously [Fig. 3D and Fig. 5C of https://doi.org/10.1242/dev.154609; Fig. 3 of https://doi.org/10.1088/1478-3975/ab6356]. Also, the diversified phenotypes within wild-type nematode embryos [Fig. 7 of https://doi.org/10.1242/dev.200401] or RNAi-treated nematode embryos [Fig. S10 of https://doi.org/10.1371/journal.pcbi.1009755] are not surprising, where experimental scientists usually only consider the highly reproducible phenotypes as the unreproducible ones can be from all kinds of unknown reasons.

We strongly agree with the reviewer’s view of this phenomenon. Changes in the concentration of one input into a nonlinear system (for example changing the concentration of a protein in a regulatory network) can induce diverse phenotypes that are not simple linear mappings of the change, as we show examples of in Figure 4B (i and ii). In the case of RNAi screens, this outcome is often simplified under the umbrella of “incomplete penetrance”, which here we demonstrate is an oversimplification of the underlying biology as RNAi treatments against the same gene in which the embryo is ultimately non-viable manifest phenotypes that are not only distinct from the wild-type organization of the embryo

but are divergent, often in a structured manner, between RNAi treated embryos. This is discussed in the manuscript on line 295 of page 11.

We respectfully disagree with the reviewer's assertion that variable phenotypes are uninformative in experimental biology. Variability, both as an input to a normal system or as a consequence of a perturbed system, is an important phenomenon in biology. Noisy responses to stimuli have been repeatedly shown to confer advantageous properties to adaptive systems such as robustness or hypersensitivity. The noise properties of systems under perturbation have similarly been used to derive insights into the underlying dynamics. In this sense, the use of robust metrics to describe biological phenomena and capture the quantitative properties of variability inherent to biological systems is an important and useful area of research.

Specific Comment 12:

12. Page 35, Line 708, "Biophysical parameters such as cell volume affect cell cycle duration…" —— There are two other pieces of literature that quantitatively measure how cell volume affects cell cycle duration during C. elegans embryogenesis [https://doi.org/10.1088/1367-2630/aaea91; https://doi.org/10.1103/PhysRevE.104.054409], [https://doi.org/10.3389/fphys.2014.00529]

We recognize the prior literature on how cell volume affects cell cycle duration, noting that cell cycle time is negatively correlated with cell size https://doi.org/10.1103/PhysRevE.104.054409]. More specifically, we now note that the relationship between these two parameters is a long tailed distribution that resembles a power law. This relationship can be modeled using the biophysical parameters of cell volume and kinetics of a protein that drives cell cycle progression [https://doi.org/10.1088/1367-2630/aaea91; https://doi.org/10.1103/PhysRevE.104.054409]. [https://doi.org/10.3389/fphys.2014.00529]. These references are added to the discussion on line 816 of page 25.

Reviewer 3 Commentary:

Reviewer #3: This manuscript proposes pairwise metrics for characterizing differences between cell lineage trees and subtrees. The metrics proposed are the tree edit distance, which describes the structural difference between two trees, and the branch distance, which measures the difference between distributions of one phenotype on two trees. Branch distances are divided up into intersection and union distances where the intersection distance is restricted to parts of two trees that share the same topology.

The metrics are used to describe the variability of cell cycle times in wild type and RNAi-treated C. elegans lineage trees. Notch signaling is manifested in the shortening of cell cycle times and a distinctive pattern of branch distances between certain lineages, a pattern which is disrupted in embryos treated with RNAi against glp-1.

The authors also develop a heuristic to describe the proximity of cell cycle times between a homeotically-transformed lineage and wild-type lineages. Here samples of wild-type lineages are used to describe the neighborhood of the destination lineage to account for variability between wild-type embryos. They show that for a few genes, RNAi treatment results in cell cycle times in the transformed lineage match those in the destination lineage. Their results quantify the considerable variability in penetrance and severity for these

genes.

This paper makes the argument that developing a set of measures for describing the distribution of phenotypes on cell lineage trees will provide quantitative insights into the mechanisms of development, and in fact other cellular processes. I strongly agree. I do have some minor questions and suggestions to improve clarity:

Comment 1:

p.4 line 86: "the distinct problem in comparing phenotypic measurements aligned to lineages has been less extensively explored." Can you give any references?

> We note that much of the work of development in *C. elegans* categorizes phenotypes in specific developing cells by taking pairwise summary statistics of regression based methods. We thus introduce the branch distance on the topology of the lineage itself and thus introduce a method to compare lineages without the use of regression based analyses. The branch distance is a rigorously defined metric and thus allows for distance based comparisons that preserve ideas of proximity and neighborhood, while making use of the binary tree topology of the lineage. This is clarified in the revised manuscript on line 87 of page 4.

Comment 2:

p.7 line 156: In the union branch distance, a 0 is imputed for cell positions which are not shared between the two trees. Can you justify this?

> If two nodes in separate trees share the same label, each embryo has measurements in the same basis vector, which is represented by a cell. However, if one tree has a node that another one doesn't, this is analogous to one tree having a measurement in a particular basis that the other tree doesn't have a measurement in. This means that this node weight contributes fully to the distance vector by squaring it, noting that a shared node would only contribute the difference between the measurements squared. Functionally, this is equivalent to imputing a zero value for any missing nodes. We argue this choice best reflects the nature of the branch distance as a distance metric. This has been clarified on line 162 of page 5 of the revised manuscript.

Comment 3:

p.10 Fig 2: Can you show the line y=x in Figs 2A and 2D? This would be a helpful guide to the eye for the discussion about slopes on p.12. (An even more trivial suggestion is to swap the positions of Figs 2C and 2D to align with the arrangement in Fig 2A and 2B.)

> We thank the reviewer for this suggestion, and have added a line of slope m = 1 to provide a reference in all subcomponents of figure A.

Comment 4:

p.11 Fig 3A: I am puzzled why there appears to be 21 embryos in the larger batch (and 9 in the smaller batch). Yet the discussion on p.20 consistently refers to the larger batch having 22 embryos.

We appreciate your noticing this discrepancy. It has been fixed such that the larger batch is noted as having 21 embryos in all analyses. In addition, we have re-run all analysis that may have been affected by this and updated the relevant data.

Comment 5:

p.13, Fig 4B: The color bars are too small.

To improve visual clarity, we have made the color bars in figure 4B larger.

Comment 6:

p.14, Fig 4 caption: It is not clear that the 30 WT embryos belong to cluster 3. Can this be stated explicitly in the caption or main text? I understand this is made clear in S1 and the S1 dataset. Also, it appears that embryos treated with a specific RNAi type are not necessarily grouped into the same cluster. Can you state this explicitly?

We have made the grouped 30 WT embryos presence in cluster 3 more clear by explicitly annotating Figure 4A cluster 3 with a WT label.

Comment 7:

p.17, Figs A, C, D: Do the fine-grained striations in these figures represent different embryos? I assume the cells from each lineal position from each embryo are grouped together on the plot.

In Figure 5, we see each fine-grained striation as representing different lineages from different embryos. The identity of the lineages is listed below each plot as the label of the identity of the progenitor cell. The cells representing the ancestor of each lineage are grouped together on each of the plots. We have clarified this point in the caption of Figure 5, on line 347 of page 13 of the revised manuscript.

Comment 8:

p.22, line 381: “In some cases, this value is 0 but overlaps the WT origin lineage neighborhood (green square) suggesting that the RNAi perturbed lineage maintained its original fate, at least in terms of cell cycle timing.” This seems to suggest that your transformation efficiency heuristic cannot distinguish between unperturbed lineages and perturbed lineages since they both have a transformation efficiency of 0?

We note that the transformation efficiency looks at neighborhoods of *C. elegans* sublineages, using the fact that similar sublineages have shorter branch distances between embryos. These neighborhoods are often clearly distinguishable, as branch distances within groups are significantly different from branch distances between groups with the sole exception being that ABplp is not distinguishable from ABprp. This is used to distinguish between unperturbed and perturbed lineages, since perturbed lineages have no WT neighbors and unperturbed lineages have WT origin neighbors. This is a separate measurement, but similar in principle to the transformation efficiency, which only counts WT destination neighbors. The transformation efficiency heuristic we describe thus does not distinguish between perturbed and unperturbed lineages as you observe. Our decision to focus on the transformed cases

here stems from our ability to interpret those observations based on the function of the genes whose knock-down generates homeotic transformations in fate. We summarize the frequency of perturbed cases across the dataset in Figure 6C but are not able to derive further insight into these phenomena other than to say that it is a common outcome of RNAi treatment against developmental regulators. We have clarified this point in the caption of Figure 6, on line 417 of page 16 of the revised manuscript.

Comment 9:

p.22: If I understand this argument correctly, you are only focused on the destination lineage as defined by the fate markers from Du et al. If the cycle time vector happens to be in the neighborhood of another lineage that is not the destination lineage, would you still say that it has no neighbors?

We focused on cases where the branch distance draws correspondences between lineages that have genetic evidence for inferring a transformation in fate based on the characterization of fate markers by Du *et al.* We have performed the unbiased search you describe and listed lineages with neighbors that are not the WT destination lineage based on fate marker matching in Supplemental Dataset (S4). This has been added in the revised manuscript on line 473 of page 18.

Comment 10:

p.22, line 398: Is a transformation score the number of neighbors up to a maximum of 22? And the transformation efficiency is the score divided by 22?

We would like to clarify that the transformation score is the number of WT neighbors of a particular destination lineage (up to 21), divided by the total number of WT embryos that it is possible to be a neighbor to (21). This is mentioned in line 440 of page 17 of the revised manuscript.

Comment 11:

p.23, line 415: You might clarify the sentence "(Fig 6B, 6C) noting a region of high density around WT neighborhoods (S7 Fig)."

We have, in Figure 6B, found the number of WT destination neighbors of homeotic transformations and in Figure 6C, found the number of WT neighbors of all sublineages of RNAi sublineages. In each of these cases, most RNAi sublineages have no neighbors. However, of the RNAi sublineages that do have neighbors, most sublineages tend to have all 22 possible wild types than not. This implies that a region of high density of RNAi lineages exists around the WT lineages, as many lineages with knocked down genes tend to cluster very close to the WT neighborhoods. We have clarified this sentence in the revised manuscript, on line 456 of page 17.

Comment 12:

p.24, line 432: Can you be more explicit about how your transformation efficiency/score relates to penetrance and phenotypic severity?

If many RNAi embryos for a specific gene have WT destination neighbors (number of embryos with nonzero transformations scores) then we call that penetrance, as it seems the RNAi treatment consistently produced a destination phenotype. If RNAi embryos that have WT destination neighbors have many destination neighbors (embryos with high transformation scores), then we call that severity,

as the RNAi treatment that did occur corresponds as closely as is possible with the destination phenotype. As such, the transformation score for each lineage is a measure of phenotypic severity, while looking at the consistency of the transformation score across various embryos can be thought of as a measure of RNAi penetrance. We have clarified this point in the manuscript on line 478 of page 18.

Comment 13:

p.24, line 433: When you say penetrance and severity “are separable phenomena” do you mean “can be distinguished”?

As penetrance relates to the number of RNAi lineages that have transformed, while severity relates to the degree to which an individual lineage has transformed, we note that these two are distinct phenomena which are not always correlated. Since each can be distinguished, we note that the underlying mechanisms that control penetrance and severity may be separate. This is specified on line 476 of page 18 of the revised main text.

Comment 14:

p.25, line 467: GITHUB reference has no address or link.

We have added a functional GitHub link to the final manuscript, at line 551 of page 20.

Comment 15:

p.31, line 607: Can you be more explicit about what you are randomizing? Is it the cells between all positions and embryos or just between embryos but at the same lineal position?

We would like to clarify the process that we used to assign random cell cycle times to cells. We are randomizing cell cycle times between cells using only the values that were present within the same embryos. This shuffling thus selectively destroys only the lineage-aligned nature of the cell cycle times while keeping all other population-level features of each embryo constant. We have clarified this point in the manuscript on line 648 of page 22.

Comment 16:

p.32, line 643: This was quite confusing. Can you state the null model that is being tested by this type of randomization?

The vast majority of homeotically transformed lineages have ~ 90 lineages with any neighbors and ~ 15 lineages with 21 neighbors. In the set of all RNA knocked down sublineages, ~700 lineages have 21 neighbors and ~3000 have 1 or more WT neighbors. In order to compute the probability of detecting a homeotic transformation between a pair of lineages due to pure chance, we assigned each cell in each RNAi embryo a random cell cycle time from the same embryo. This preserves the embryo-specific distribution of cell cycle times but randomizes their assignment to specific positions with that embryo’s lineage. Using embryos randomized in this manner, we calculated the number of WT neighbors of each lineage in these shuffled embryos. This bootstrapping procedure provides a baseline, allowing us to see the degree to which randomized lineages would match WT lineages. This is mentioned in line 745 of page 24 of the revised manuscript.

Comment 17:

p.35, line 693: Where were the “linear gradients of severity” described?

This phrase refers to the fact that knocking down a particular gene can produce a spectrum of phenotypes in certain embryos of variable severity. In measuring the severity of those phenotypes in divergence from the WT through the branch distance or Tree Edit Distance we find that knocked down genes produce a variety of branch or tree edit distances. In this case, we find that these branch/tree edit distances follow a gradient of severity of the WT, that seems to have a linear relationship. This is found in Figure 4Biii and 4Biv in measuring SKR-2 RNAi embryos. We have added additional text to the manuscript to describe this observation, on line 799 of page 25 of the revised manuscript.

Comment 18:

p.44: I found myself constantly flipping to the supporting figures. I would consider incorporating some of these into the main text.

We appreciate the feedback. To help with organization, we have decided to consolidate supplemental figures 4 and 5. We feel that the manuscript, as it currently stands, is suitably long. Incorporating supplemental figures would impair narrative cohesion, and the succinctness of our writing. Thus, we would defer to the preferences of the reviewers and the editor as to whether to reposition supplemental figures into the main text.

Comment 19:

S2: I found it very difficult to read the labels in this figure. Can the pixelation be made to have higher resolution?

We have remade the labels in this figure with a higher resolution.

Comment 20:

S3: Should the y axis say “mean branch distance” instead of “mean branch edit distance”?

We have corrected the label on this figure.
